# Increased AOC1 Expression Promotes Cancer Progression in Colorectal Cancer

**DOI:** 10.3389/fonc.2021.657210

**Published:** 2021-05-05

**Authors:** Fangyuan Liu, Weijun Ou, Wenbo Tang, Zhenyu Huang, Zhehui Zhu, Wenjun Ding, Jihong Fu, Yilian Zhu, Chenying Liu, Weimin Xu, Peng Du

**Affiliations:** Department of Colorectal Surgery, Xinhua Hospital, Shanghai Jiaotong University School of Medicine, Shanghai, China

**Keywords:** *AOC1*, colorectal cancer, proliferation, migration, organoid, EMT

## Abstract

**Background:**

Amine oxidase copper containing 1 (*AOC1*) is a gene whose biological function in colorectal cancer (CRC) has not been elucidated. Therefore, the purpose of this study was to investigate the clinical significance of *AOC1* expression in CRC and its biological function in CRC cell lines.

**Materials and Methods:**

*AOC1* expression levels were examined in paired CRC and peritumoral tissues, and distant liver metastatic tissues were examined using quantitative real-time PCR, western blotting, and immunohistochemistry staining. The log-rank test and Cox regression model were used to analyze the relationship between *AOC1* expression and prognosis. Proliferation assays (Cell Counting Kit‐8 and colony formation assays), migration assays (Transwell and wound healing assays) and xenograft tumor formation in nude mice were performed to assess the biological role of *AOC1* in CRC cells.

**Results:**

*AOC1* expression significantly increased in human CRC tissues, especially in liver metastases, and was associated with a worse prognosis. In addition, *AOC1* had higher expression in tumor organoids than in normal organoids, suggesting that it was highly expressed in the tumor epithelium. Functional analysis demonstrated that *AOC1* knockdown inhibited the proliferation and migration of CRC cells by inducing EMT *in vitro*. Xenograft tumor formation in nude mice showed that knockdown of *AOC1* inhibited the tumor xenografts growth *in vivo*.

**Conclusion:**

High expression of *AOC1* was significantly associated with worse clinical outcomes, was an independent risk factor for poor prognosis, and promoted aggressive CRC cell phenotypes. *AOC1* is expected to become a novel biomarker for predicting the prognosis of patients with CRC and an effective therapeutic target in clinical practice.

## Introduction

Colorectal cancer (CRC) is the second most common malignant tumor, with the fourth highest mortality rate. Annually, approximately 1.25 million people worldwide are diagnosed with CRC and more than 600,000 patients die from it (1). In the past 20 years, the incidence rate of CRC has increased significantly, especially in larger cities, and there has been a trend of younger disease (2, 3). Therefore, CRC has become a major global health concern. Despite advancements in treatment in the past few decades, the mortality rate of CRC is still high, mainly due to recurrence and distant organ metastasis (4). Twenty percent of CRC cases have metastases, mostly in the liver (5, 6). Although some diagnostic biomarkers, such as CEA and CA199 (7), are established, it is still necessary to explore new molecules to accurately predict the prognosis of CRC patients and become effective therapeutic targets in clinical practice.

*AOC1* (Amine Oxidase Copper Containing 1) is a protein coding gene and it encodes a metal-binding membrane glycoprotein that oxidatively deaminates putrescine, histamine, and related compounds. Previous studies have shown that polyamines are involved in the regulation of cell migration, proliferation, and apoptosis.

Recently, a study (8) revealed that *AOC1* promoted the progression of gastric cancer, and another study (9) has shown that *AOC1* was a downstream target gene of the Wilms tumor protein that affected kidney development. However, very little is known about the functions and regulatory mechanisms of *AOC1* in CRC.

In the current study, we detected the expression of *AOC1* in CRC tissues and found that *AOC1* expression was significantly increased in human colorectal cancer tissues compared to normal tissues. In addition, the expression was higher in liver metastases than in carcinoma in situ. Furthermore, with the arrival of the era of organoid culture *in vitro* (9), we used organoids in the experiment to verify the related phenomenon. As we had expected, *AOC1* was highly expressed in tumor organoids compared to the normal one, suggesting that it was highly expressed in the tumor epithelium. We first reported the biological functions of *AOC1* in CRC cells, especially its effects on proliferation and migration. Moreover, *AOC1* overexpression promoted the proliferation and migration of CRC cells. Consistent with the result of the cell proliferation assay *in vitro*, knockdown of AOC1 significantly inhibited the tumor xenografts growth. Interestingly, *AOC1* knockdown inhibited the migration and proliferation of CRC cells by EMT pathway. Therefore, *AOC1* is expected to become a novel biomarker for predicting the prognosis of patients with CRC and an effective therapeutic target in clinical practice.

## Materials and Methods

### Patients and Specimens

From January 2010 to January 2013, the Department of Colorectal Surgery, Xinhua Hospital, Shanghai Jiaotong University School of Medicine, admitted 192 CRC patients. Formalin-fixed, paraffin-embedded (FFPE) tumor tissues and matched normal tissues were used to make a tissue microarray (TMA) for further immunohistochemistry (IHC) analysis.

Thirty fresh paired tissues were analyzed by real-time quantitative polymerase chain reaction (qRT-PCR), and four fresh paired tissues were analyzed by western blotting. The Ethics Committee of Xinhua Hospital approved this study (No. XHEC‐D‐2021‐002). All patients enrolled in this study signed a broad consent form, and the study was strictly in accordance with the Declaration of Helsinki and International Ethical Guidelines for Health-related Research Involving Humans.

### IHC and Histopathologic Evaluation of *AOC1* Expression

The TMA and xenograft tumor sections was deparaffinized, followed by antigen retrieval using citrate buffer (pH 6.0). Hence, to block endogenous peroxidases and nonspecific antigens, 3% hydrogen peroxide, and 5% goat serum were used, respectively. The primary antibody against *AOC1* (1:500, Abcam) and anti‐Ki67 (1:1,000, Catalog No. ab16667; Abcam, Boston, MA) was incubated overnight at 4°C. The secondary antibody was then applied to the TMA or xenograft tumor sections for 1 h at room temperature after washing with phosphate-buffered saline (PBS) three times for 5 min each. Finally, to detect the positive expression of the primary antibody, diaminobenzidine chromogen (Beyotime, Haimen, China) was performed. The TMA was then counterstained with hematoxylin and cover slipped.

Excluding patients who failed to follow-up, 151 eligible patients were enrolled in this study. Immunohistochemical analysis was conducted as previously described. *AOC1* expression in TMA was evaluated and semi-quantitatively scored based on IHC results by two independent pathologists. Semi-quantitative IHC analysis was scored based on staining intensity and percentage of staining. The detailed standards were as follows: staining intensity: 0 (negative), 1 (weakly positive), 2 (moderately positive), or 3 (strongly positive); percentage of staining: 1 (<25%), 2 (25% - 50%), 3 (50% - 75%), and 4 (> 75%). Expression index = % of positive cells × staining intensity. The *AOC1* protein expression in CRC specimens was divided into the low expression group (< 4) and high expression group (≥ 4) for subsequent analysis.

### Cell Culture and Treatment

Human CRC cell lines, including HCT116, LoVo, sw480, HT29, and HEK293T cells were obtained from the American Type Culture Collection (Manassas, VA, USA). All cells tested negative for mycoplasma contamination before use. Cells were cultured in Dulbecco’s modified Eagle’s medium (DMEM; HyClone, Los Angeles, CA, USA) supplemented with 10% fetal bovine serum (FBS; Gibco, Grand Island, New York, USA), 100 µg/mL of penicillin, and 100 U/mL of streptomycin at 37°C with 5% CO_2_.

### Organoid Culture

Fresh tumor and paired adjacent normal colon tissues were kept in phosphate-buffered saline solution without calcium and magnesium (PBS WO, Sigma Aldrich) at 4°C. The isolation of healthy crypts and tumor epithelium was performed as described by Sato et al. (10). The organoid culture medium was refreshed every two days. Culture medium including 50% L-WRN condition medium (containing Wnt3a, R-Spondin1, and Noggin), 1X Glutamax, 1X hepes, 1X N2 (Invitrogen), 1X B27 minus vitamin A (Life Technologies), 1X penicillin/streptomycin solution (Invitrogen), 50 ng/mL human EGF (Gibco), 1 mM N-acetylcysteine (Sigma Aldrich), 10 mM nicotinamide (Sigma Aldrich), 10 nM gastrin (Sigma Aldrich), 10 µM SB202190 (Selleck), 0.01 uM PGE2 (Sigma Aldrich), and 10 mM Y27632 (Sigma Aldrich).

### RNA Extraction and RT‐qPCR

Total RNA was extracted from the cultured cells and fresh tissues using TRIzol reagent (Invitrogen, Carlsbad, CA, USA), according to the manufacturer’s instructions. To reverse the RNA, the PrimeScript ™ RT Master Mix (Takara Biotechnology Co, Ltd.) was used. The SYBR Premix ExTaq ™ (Takara, Japan) and an Applied Biosystems 7500 Fast Real-Time PCR System (Applied Biosystems, Waltham, MA) were used for qRT-PCR measurements. Relative mRNA expression levels were evaluated using the 2 −ΔΔCt method and normalized to the expression of β-actin. All experiments were performed in triplicate.

The sequences of the PCR primers applied are listed as:

AOC1-F: CCTAAGCAACCAAGAGCTGAA
AOC1-R: CGGTGACATTGGGATGCTCC
Actin-F: GCACAGAGCCTCGCCTT
Actin-R: GTTGTCGACGACGAGCG.

### Western Blotting

Cultured cells, fresh tumor tissue, and paired adjacent normal colon tissues were lysed with 1% NP40 lysis buffer supplemented with NaF, Na_3_VO_4_, and protease inhibitor cocktail. Lysates containing equal amounts of protein (20 μg/well) were loaded and separated by SDS-PAGE and then transferred onto a nitrocellulose membrane. The membrane was blocked with 5% non-fat milk for at least 1 h and then incubated with a specific antibody against *AOC1* (1:500, Abcam) at 4°C overnight. After washing the membranes thrice with Tris-borate saline containing 0.1% Tween-20 for 5 min each, the specific protein was visualized with horseradish peroxidase (HRP)-conjugated secondary antibody and enhanced chemiluminescence.

### RNA Interference

Short hairpin RNA (shRNA) sequences targeting *AOC1* were cloned into a pLKO vector with a pMD.2G and psPAX2 packaging system, and lentiviruses were generated in HEK293T cells. To transfect this vector, sw480 cells were infected with viral particles and treated with PB (polybrene) to promote transfection efficiency. Then, stably transfected cells were filtered for 72h using puromycin. RT-qPCR was used to analyzed the knock-down efficiency.

The shRNA sequences are as follows:

shAOC1-1F: GCGGACAACTTCAACTGTCTA
shAOC1-1R: TAGACAGTTGAAGTTGTCCGC
shAOC1-2F: CCTAAGCAACCAAGAGCTGAA
shAOC1-2R: TTCAGCTCTTGGTTGCTTAGG

### Enforced Expression of *AOC1* in CRC Cell Line HCT116

The cDNA of *AOC1* was cloned into a puromycin-resistant lentiviral vector (pLVX-Puro). Lentivirus particles were prepared using HEK293T cells according to the manufacturer’s protocol (Takara, Beijing, China). HCT116 cells were then seeded into six-well culture plates and incubated overnight. They were subsequently infected with *AOC1*-expressing lentivirus, treated with PB to promote transfection efficiency, and then treated with puromycin to select the cells overexpressing *AOC1* in a stable manner.

### Cell Counting Kit‐8 and Colony Formation Assays

The CCK8 assay was employed to detect the effect of *AOC1* knockdown on cell proliferation. First, a cell count of approximately 1,000 cells/well was plated on five 96‐well plates, and five replicates were cultured with complete medium (DMEM with10% FBS). After incubation for 24 h, 10 uL/well CCK‐8 solution was added to each well in the first 96‐well plate and incubated for 1.5 h at 37°C in dark conditions. A microplate reader was used to detect the absorbance at 450 nm in each plate for 5 days consecutively.

For the colony formation assay, approximately 1,000 cells were plated in 6-well plates and then cultured in DMEM containing 10% FBS. After 2 weeks, the cells were washed with PBS and fixed with 4% paraformaldehyde for 30 min at room temperature. Finally, 0.1% crystal violet was used to stain these cells for 30 min at room temperature and then washed off by clean water. The number of cells was then observed in each cell group.

### Transwell and Wound—Healing Assays

To assess the effect of *AOC1* overexpression and knockdown on cell migration ability, a transwell assay was performed. Subsequently, stable *AOC1*-overexpressing and control cells (1.0 × 10^5^) were harvested and suspended in 100 uL serum-free medium and seeded into the upper chamber, while the lower chambers were filled with 500 μL complete medium and incubated for 65 h. The upper chamber was fixed with 4% paraformaldehyde for 30 min at room temperature. Finally, 0.1% crystal violet was used to further stain these cells adhering to the upper chamber for 30 min at room temperature. Cell migration ability was assessed based on the number of cells passing through the upper chamber.

To verify the effect of *AOC1* knockdown on tumor cell migration ability, we used the aforementioned method to change the cell mass to 1.5 × 10^5^ *AOC1* knockdown stable cells, with an incubation time of 48 h.

In addition, a wound-healing assay was performed to confirm the effect. A total of 1 × 10^6^ cells were seeded into six-well plates and cultured in low-serum medium (DMEM containing 1% FBS). When the cell density was 100%, a scratch wound was made in the cell monolayer, and the initial image was immediately obtained. The cells were then cultured for another 48 h, 72 h, and 96 h, and the corresponding images were recorded immediately as well.

### Xenograft Tumor Mice Model

All animal experimental procedures were approved by the Laboratory Animal Care and Welfare Committee of Xinhua Hospital (No. XHEC‐F‐2021‐057). To study the effect of AOC1 on CRC cells proliferation *in vivo*, 10 male nude mice (4-6 weeks old) were used as the experimental animals. Among the 10 nude mice, 5 were injected with AOC1-depletion CRC cells, and the another 5 nude mice were used as a control group. First, 1 × 10 6 sw480pLKO and sw480 shAOC1cells were counted and then subcutaneously injected into both axillary fat pads in a different group. Then these nude mice were fed for consecutive 2 weeks until the last day. Tumors were removed from killed mice, photographed, and paraffin preserved (11).

### Statistical Analysis

GraphPad Prism 8 Software (GraphPad, San Diego, CA) and SPSS version 26.0 software (IBM, 2010, Chicago, IL) were used for overall statistical analyses. Kaplan–Meier method was performed to assess the survival time distribution, and the log‐rank test was used to test significance in DFS (Disease free survival) and OS (Overall survival) among the different prognostic groups. The Cox proportional hazard model was used perform single and multivariate analyses to evaluate the hazard ratio (HR) and 95% confidence interval (CI). In our previous studies, we described the detailed statistical methods (12, 13). Unless otherwise stated in the legend, ANOVA or two-tailed Student’s t-test was used to assess significance. The chi-square test was used to analyze the correlations of AOC1 expression with clinicopathological characteristics of CRC patients. Three biological replicates were performed for each experiment. A p-value less than 0.05 was considered statistically different (*P < 0.05; **P < 0.01; ***P < 0.001).

## Results

### Increased *AOC1* Expression in Tumor Tissues Was Associated With Worse Prognosis in CRC Patients

We included 151 eligible CRC patients in the current study, and the median follow-up time from January 2015 to July 2020 was 57.0 (17.0 - 71.0) months. **Table 1** shows the demographic, laboratory, and clinical characteristics of the patients.

**Table 1:** Baseline characteristics of patients.

Variable	Patients
**Sex ( M/F )**	79/72
**Age [yr, median (IQR)]**	66.0 (55.0-76.0)
**Follow up [mo, median (IQR)]**	57.0 (17.0 -71.0)
**Tumor Site, n (%)**	
Rectum	30 (19.9)
Left side	53 (35.1)
Right side	68 (45.0)
**Histology, n (%)**	
Well differentiated	15 (9.9)
Moderately differentiated	108 (71.5)
Poorly differentiated	28 (18.5)
**T-stage, n (%)**	
T1	2 (1.3)
T2	15 (9.9)
T3	51 (33.8)
T4	83 (55.0)
**N-stage, n (%)**	
N0	76 (50.4)
N1	47 (31.1)
N2	28 (18.5)
**Cancer stage, n (%)**	
I	13 (8.6)
II	62 (41.1)
III	64 (42.4)
IV	12 (7.9)
**Serum albumin, n (%)**	
≥35 g/L	125 (82.8)
<35 g/L	26 (17.2)
**Hemoglobin, n (%)**	
≥110 g/L	106 (70.2)
<110 g/L	45 (29.8)
**Serum carcinoembryonic antigen (CEA), n (%)**	
<10 ng/ml	104 (68.9)
≥10 ng/ml	47 (31.1)
**AOC1 expression status, n (%)**	
Low expression	74 (49)
High expression	77 (51)

F, female; M, male; AOC1, Amine Oxidase Copper Containing 1; IQR, interquartile range.

**Figure 1:**
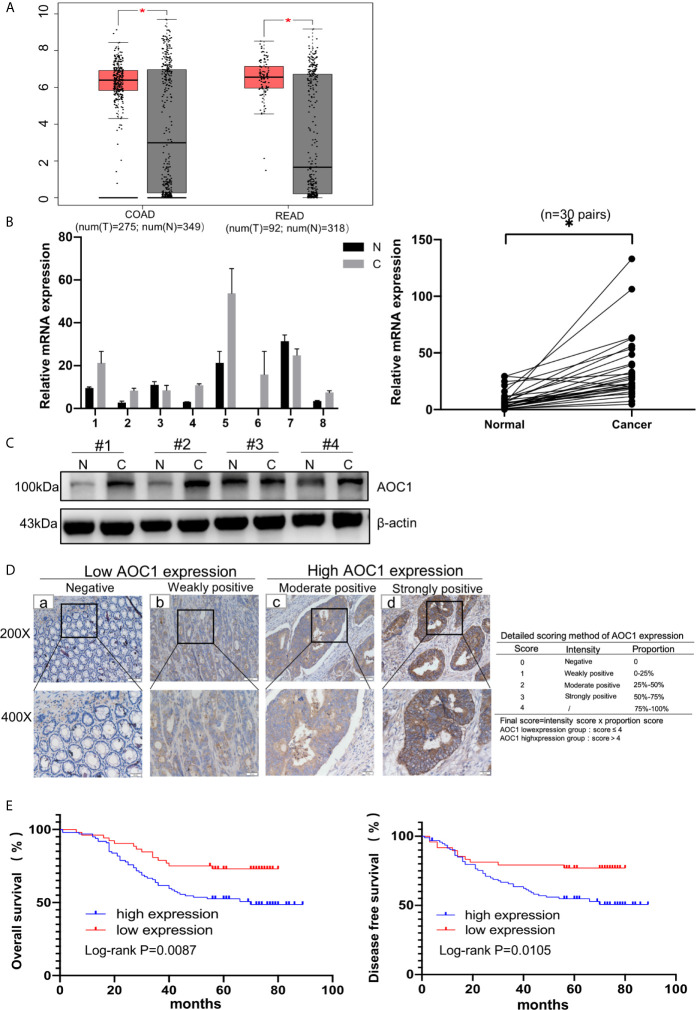
*AOC1* expression was increased in tumor tissues and associated with worse prognosis. **(A)** Data were obtained from the GEPIA website, *AOC1* was highly expression in tumor tissue (red box) compared with normal tissue (gray box), both in COAD and READ (14). **(B)** Tumor and paired normal colorectal tissues were detected the *AOC1* expression by real‐time quantitative polymerase chain reaction and Western blotting **(C)**. **(D)** Immunohistochemical analysis of different *AOC1* expression in CRC patients (magnification: × 200, Low; × 400, High)and detailed scoring method of *AOC1* expression; Da‐Dd represented negative, weakly positive, moderate positive, and strongly positive *AOC1* expression, respectively. **(E)** The Kaplan–Meier plots were stratified by *AOC1* expression for disease‐free survival and overall survival in CRC patients. Log‐rank test was performed to assess statistical significance. CRC, colorectal cancer; *AOC1*, Amine Oxidase Copper Containing 1; mRNA, messenger RNA; COAD, Colon adenocarcinoma; READ, Rectum adenocarcinoma. OS, overall survival; DFS, disease-free survival. *P < 0.05.

First, we found AOC1 was highly expression in tumor tissues both in Colon adenocarcinoma (COAD) and Rectum adenocarcinoma (READ) (**Figure 1A**), according to Gene Expression Profiling Interactive Analysis (GEPIA) database (14). we tested the mRNA and protein levels of *AOC1* expression in paired tumor and normal tissues by qRT-PCR and western blotting, respectively (**Figures 1B, C**). These indicated that *AOC1* was highly expressed in the tumor tissues. Furthermore, immunohistochemistry (IHC) was performed on 192 CRC tumor tissues and paired adjacent normal colon tissues from Xinhua Hospital in TMA, and 41 patients were excluded from further analysis as they were lost to follow-up. As presented in **Figure 1D**, representative images of different levels of expression of *AOC1* have been displayed, including negative (Da), weakly positive (Db), moderately positive (Dc), and strongly positive (Dd). **Figure 1D** showed a detailed scoring method for assessing *AOC1* expression intensity. In the entire cohort, participants were divided into *AOC1* low expression and high expression groups corresponding to 74 samples (49%), and 77 samples (51%), respectively (**Table 1**). Moreover, we analyzed the correlations between the expression of *AOC1* and the clinicopathological characteristics of the CRC patients. Correlation analysis showed expression of *AOC1* was significant correlation with Gender, Tumor Site, Cancer stage and Hemoglobin (p =0.012; p = 0.001, p = 0.016, p = 0.031), especially with liver metastasis (p < 0.001), however, it is not associated with Histology, T-stage, N-stage, N-stage, and Serum carcinoembryonic antigen (CEA) (**Table 2**).

**Table 2:** Correlations of AOC1 expression with clinicopathological characteristics of CRC patients.

Variable	Low AOC1 expression	High AOC1 expression	P value
**Gender, n (%)**			**0.012**
Male	31(41.9)	48(62.3)	
Female	43(58.1)	29(37.7)	
**Tumor Site, n (%)**			**0.001**
Rectum	34(46)	16(20.8)	
Left side	26 (35.1)	27 (35.1)	
Right side	14(18.9)	34 (44.2)	
**Histology, n (%)**			0.532
Well differentiated	6(8.1)	9(11.7)	
Moderately differentiated	56 (75.7)	52 (67.5)	
Poorly differentiated	12 (16.2)	16(18.2)	
**T-stage, n (%)**			0.227
T1	2(2.7)	0(0)	
T2	8(10.8)	7 (9.1)	
T3	26(35.1)	25 (32.5)	
T4	38(51.4)	45(58.4)	
**N-stage, n (%)**			0.633
N0	40(54.1)	36((46.8)	
N1	22(29.7)	25(32.5)	
N2	12(17.2)	16(20.7)	
**Liver metastasis, n (%)**			**<0.001**
Presence	2(2.7)	18(23.4)	
Absence	72(97.3)	59(76.6)	
**Cancer stage, n (%)**			**0.016**
I	9(12.2)	4(5.2)	
II	32(43.2)	30(40)	
III	32(43.2)	32(41.5)	
IV	1(1.4)	11(14.3)	
**Serum albumin, n (%)**			0.587
≥35 g/L	60(81.1)	65(84.4)	
<35 g/L	14(18.9)	12(15.6)	
**Hemoglobin, n (%)**			**0.031**
≥110 g/L	58(78.4)	48(62.3)	
<110 g/L	16(21.6)	29(37.7)	
**Serum carcinoembryonic antigen (CEA), n (%)**			0.734
<10 ng/ml	50(67.6)	54(70.1)	
≥10 ng/ml	24(32.4)	23(29.9)	

Bold values indicate statistical signiﬁcance (P < 0.05); AOC1, Amine Oxidase Copper Containing 1.

**Figure 2:**
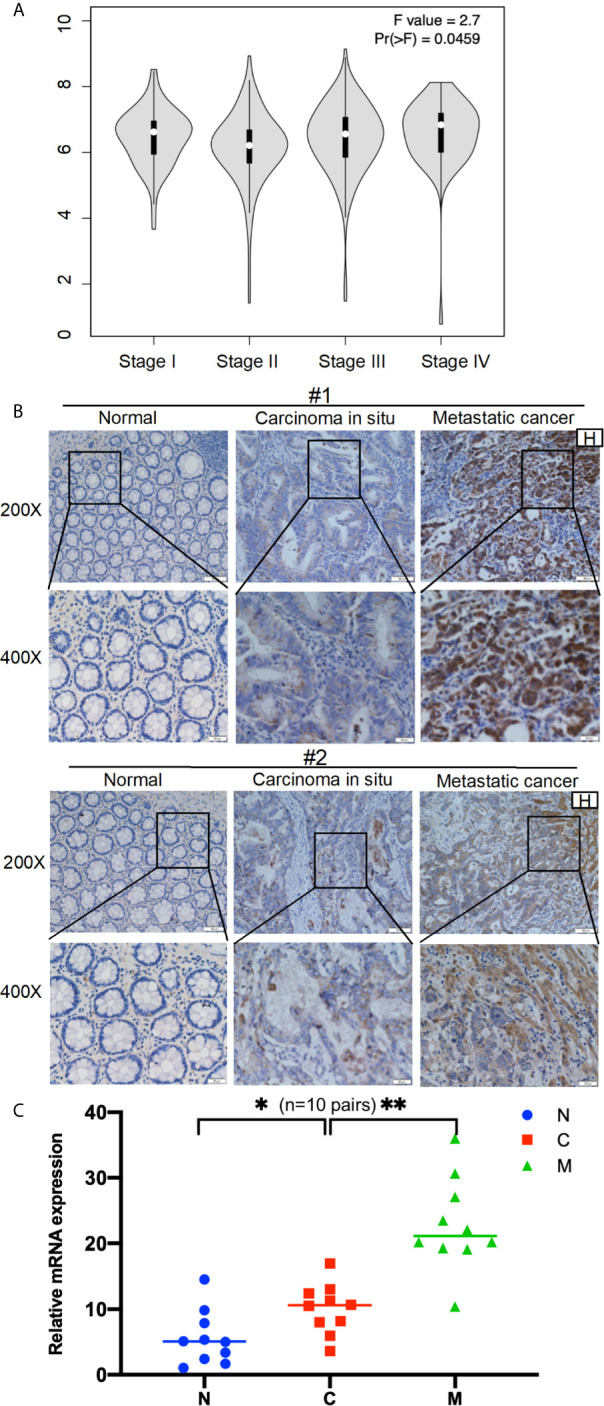
*AOC1* expression was highly increased in advanced tumor stage. **(A)** Data were obtained from the GEPIA, AOC1 expression was associated with CRC cancer stage both in COAD and READ (P=0.0459) (14). **(B)** Immunohistochemical analysis of *AOC1* expression in paired CRC tissues, peritumoral tissues and distant liver metastatic tissues (magnification: × 200, Low; × 400, High); **(C)** The expression of AOC1 in normal, tumor and liver metastases (n=10 pairs) by qRT‐PCR. H, Hepatic; *AOC1*, Amine Oxidase Copper Containing 1; COAD, Colon adenocarcinoma; READ, Rectum adenocarcinoma; qRT‐PCR, real‐time quantitative polymerase chain reaction; *p < 0.05, **p < 0.01.

**Figure 3:**
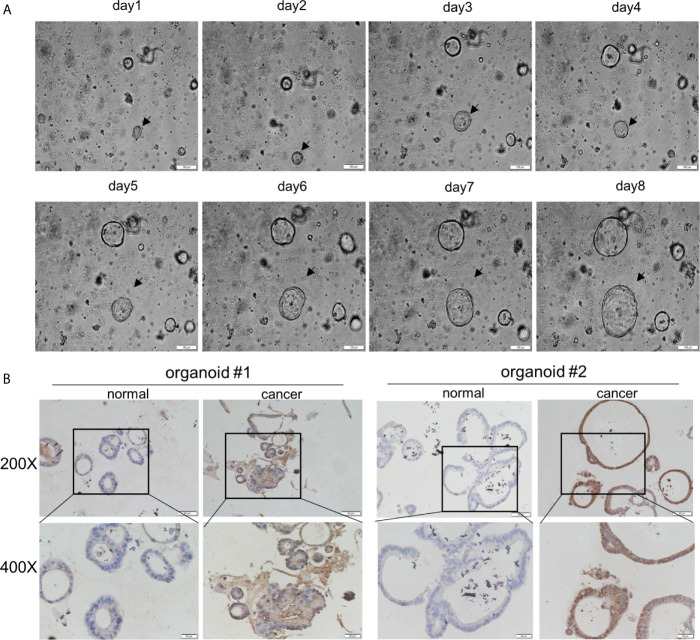
*AOC1* was highly expression in tumor epithelium. **(A)** Normal colon organoids grown from day1 to day8 (Scale bar:100um). **(B)** Immunohistochemical analysis of *AOC1* expression in normal colon organoid and tumor organoid (magnification: × 200, Low; × 400, High).

Next, the GEPIA database evaluated that *AOC1* expression was statistically significant for predicting tumor clinical stage according to GEPIA database (p = 0.0459) (**Figure 2A**). Then, we performed IHC on paired normal, tumor, and distant liver metastasis tissues. We observed that *AOC1* expression was noticeably higher in CRC liver metastatic tissues than in primary tumor tissues by IHC and qRT-PCR (**Figures 2B, C**), and significant associated with liver metastasis (p < 0.001) (**Table 2**), indicating that *AOC1* is a potential biomarker for predicting liver metastasis of colorectal cancer. To study its expression in the intestinal epithelium, IHC was performed on organoids derived from fresh tumor tissue and paired adjacent normal colon tissues. The results showed that *AOC1* was highly expressed in the tumor epithelium (**Figures 3A, B**), suggesting that the positive expression pattern of *AOC1* was the location of the tumor epithelium cytoplasm.

In univariate analysis, tumor depth, positive regional lymph nodes metastasis, clinical stage, and *AOC1* expression level were significantly associated with prognosis (p <  0.05), whereas sex, age, or primary tumor location were not associated (**Table 3**). In multivariate Cox regression hazard analysis, tumor depth, Stages‐III/IV and high *AOC1* expression were associated with poor prognosis in DFS (HR = 2.042, 95% CI =  1.200–3.475, p =  0.008; HR = 1.793, 95% CI =  1.068-3.010, p =  0.027; HR =  18.842, 95% CI =  7.459–47.597, p < 0.001), and tumor depth and high *AOC1* expression was a significant contributing factor for poor prognosis in OS (HR = 1.946, 95% CI =  1.143-3.314, p =  0.014; HR = 19.343, 95% CI =  7.649–48.920, p < 0.001) (**Table 3**).

**Table 3:** Univariate and multivariate analysis of overall survival and disease-free survival.

Variable	DFS				OS			
	Univariate	P value	Multivariate	P value	Univariate	P value	Multivariate	P value
	HR (95% CI)		HR (95% CI)		HR (95% CI)		HR (95% CI)	
**Age**		0.174				0.129		
< 65	1				1			
≥ 65	1.415(0.854-2.344)				1.472 (0.888-2.439)			
**Sex**		0.064				0.123		
Male	1				1			
Female	1.604(0.967-2.660)				1.482 (0.894-2.458)			
**Tumor location**		0.819				0.721		
Right side	1				1			
Left side	1.227(0.643-2.339)				1.279(0.671-2.440)			
Rectum	1.024(0.583-1.798)				0.989(0.563-1.737)			
**Histology**		0.129				0.096		
Well differentiated	1				1			
Moderately differentiated	0.369(0.123-1.105)				1.722 (0.618-4.799)			
Poorly differentiated	0.638(0.359-1.133)				2.851 (0.952-8.539)			
**Tumor depth**		0.005	2.042(1.200-3.475)	0.008		0.008	1.946 (1.143-3.314)	0.014
Limited under serosa (T1~T3)	1				1			
Penetrating the serosa (T4)	2.088(1.236-3.530)				1.990 (1.177-3.362)			
**Regional lymph nodes metastasis**		0.015				0.02		
Negative (N0)	1				1			
Positive (N1~2)	1.861(1.122-3.085)				1.806 (1.090-2.993)			
**Stage**		0.005	1.793(1.068-3.010)	0.027		0.005	1.642 (0.978-2.757)	0.061
I/II	1				1			
III/IV	2.064(1.235-3.449)				2.039 (1.220-3.406)			
**Serum albumin**		0.558				0.514		
<35 g/L	1				1			
≥35 g/L	1.229(0.607-2.490)				1.257(0.620-2.546)			
**Hemoglobin**		0.701				0.675		
≥110 g/L	1				1			
<110 g/L	1.110(0.653-1.887)				1.121(0.660-1.906)			
**Serum carcinoembryonic antigen (CEA)**		0.938				0.859		
<10 ng/ml	1				1			
≥10 ng/ml	1.021(0.601-1.736)				1.049 (0.617-1.784)			
**AOC1 expression status**		<0.001	18.842(7.459-47.597)	<0.001		<0.001	19.343(7.649-48.920)	<0.001
Low expression	1				1			
High expression	18.969(7.522-47.833)				19.783 (7.845-49.887)			

CI, confidence intervals; AOC1, Amine Oxidase Copper Containing 1; DFS, disease free survival; OS, overall survival; HR, hazard ratio.

In addition, in the Kaplan-Meier survival analysis using the log-rank test, patients with high *AOC1* expression had worse prognosis both in DFS (p=0.0105) and OS (p =0.0087) compared to the low-expression group (**Figure 1E**). In summary, these indicate that the upregulation of *AOC1* in tumor tissues significantly correlates with worse clinical outcomes in CRC patients and is an independent prognostic factor in CRC patients after surgery (**Table 3**).

### *AOC1* Overexpression Promoted Tumor Proliferation Ability of CRC Cell *In Vitro*

**Figure 4:**
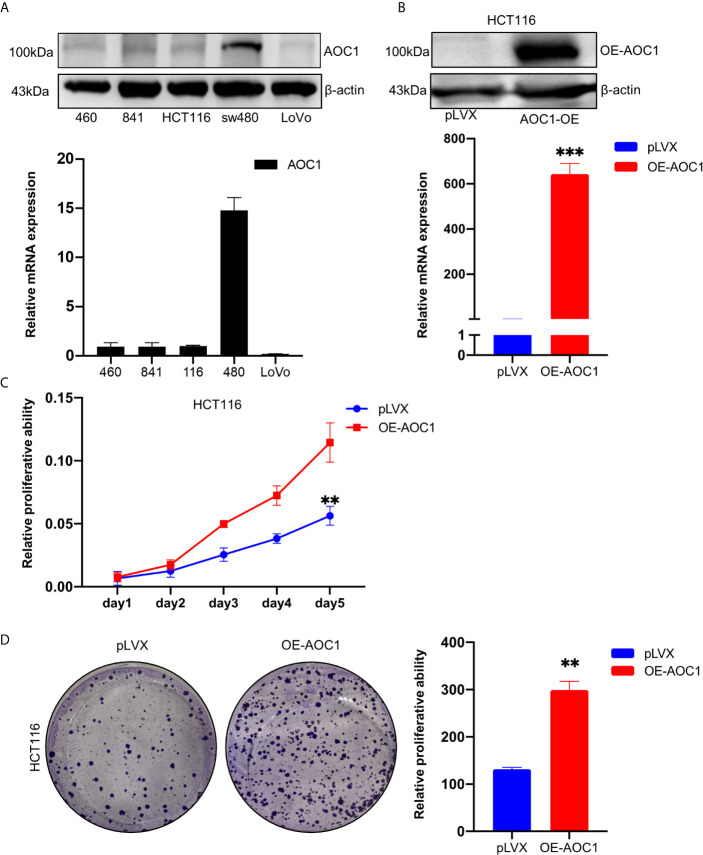
*AOC1* expression was in CRC cell lines and *AOC1* overexpression promoted the proliferation ability of colorectal cancer cells. **(A)** *AOC1* mRNA and protein level were detected by qRT‐PCR and western blot analysis in five cell lines, respectively. **(B)** The efficiency of *AOC1* overexpression were examined in HCT116 cells by qRT‐PCR and western blot analysis. **(C)** The effect of overexpression of *AOC1* on cell proliferation was detected by Cell Counting Kit‐8 assay kits in HCT116 cells. **(D)** Colony‐forming assay in *AOC1* overexpression in HCT116 cells. CRC, colorectal cancer; *AOC1*, Amine Oxidase Copper Containing 1; mRNA, messenger RNA; qRT‐PCR, real‐time quantitative polymerase chain reaction; **p < 0.01, ***p < 0.001.

To detect the expression of *AOC1*, five different cell lines including normal and CRC cell lines, were used to analyze the *AOC1* expression through qRT‐PCR and western blot analysis. As shown in **Figure 4A**, the sw480 cell line had a relatively high expression of *AOC1* both at the mRNA and protein levels, but the HCT116 cell line had a relatively low expression. To examine the effects of *AOC1* overexpression in CRC cells, HCT116 cells were selected to enhance *AOC1* expression using an *AOC1* overexpression plasmid.

Western blotting and qRT-PCR determined the efficiency of *AOC1* overexpression (**Figure 4B**). As showed in **Figures 4C, D**, we found that *AOC1* overexpression in HCT116 cells significantly promoted cell proliferation ability compared to the control cells by cck8, as well as colony formation assay. These results showed that *AOC1* played an oncogene role by promoting the malignant proliferation of CRC cells.

### *AOC1* Overexpression Promoted Tumor Cell Migration *In Vitro*

**Figure 5:**
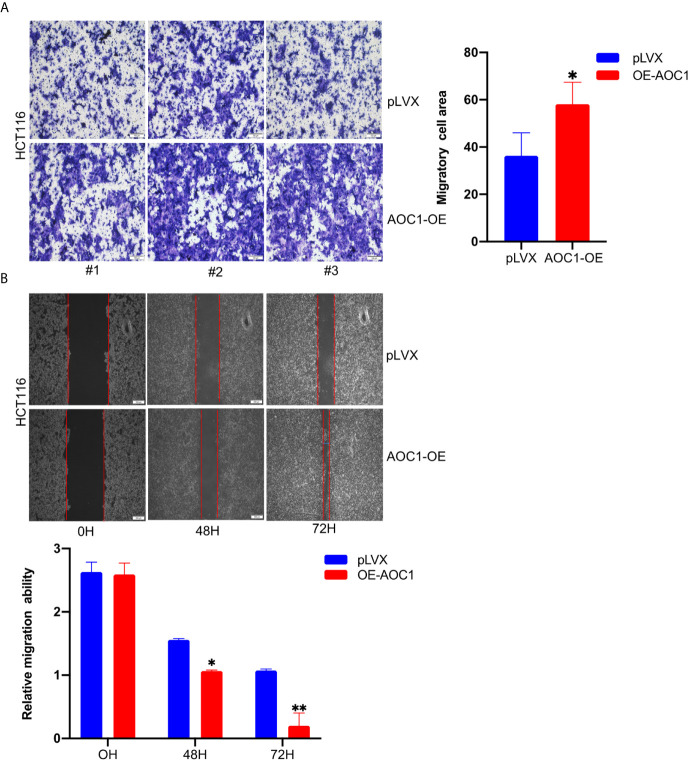
*AOC1* overexpression promoted tumor migration ability of CRC cell *in vitro*. **(A)** The effects of *AOC1* overexpression on cell migration were detected by Transwell‐migration assays in HCT116 cells (Scale bar:100um). **(B)** The migratory capacity of *AOC1* overexpression stable cells were determined by wound‐healing assay. The degree of migration was determined at 72h after the initial scratch wound (Scale bar:200um). CRC, colorectal cancer; *AOC1*, Amine Oxidase Copper Containing 1 mRNA, messenger RNA; qRT‐PCR, real‐time quantitative polymerase chain reaction; *p < 0.05, **p < 0.01.

To further analyze the effect of overexpression of AOC1 in CRC cell, the Transwell and wound-healing assays were used. As shown in **Figure 5A**, *AOC1* overexpression in HCT116 cells significantly promoted cell migration ability compared to the control cells in the Transwell assay. Furthermore, consistent results were obtained from the wound-healing assay. We found that *AOC1* overexpression in HCT116 cells also increased wound closure rates when compared with control cells (**Figure 5B**). Therefore, these results indicated that *AOC1* played a biological role in regulating the migration of CRC cells.

### *AOC1* Knockdown Inhibited the Proliferation Ability of CRC Cell *In Vitro*

**Figure 6:**
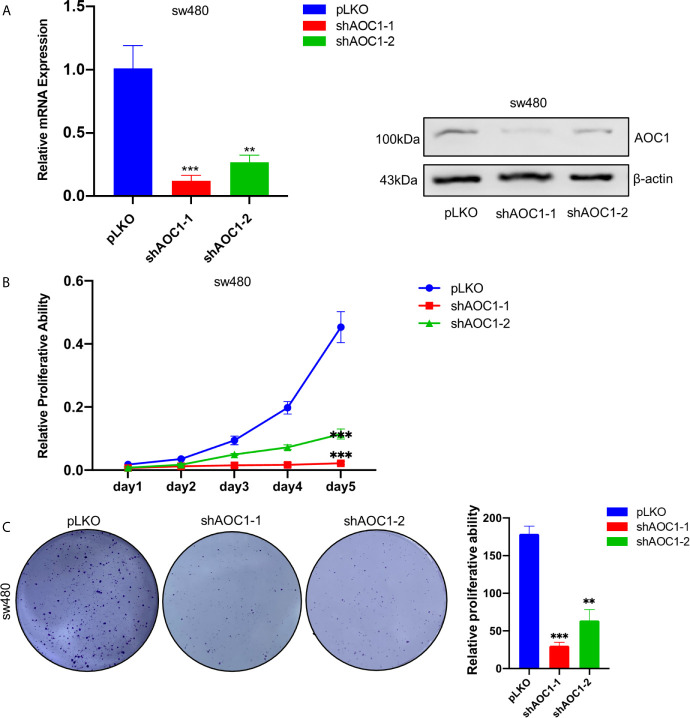
*AOC1* knockdown impaired the proliferation ability in CRC cells. **(A)** The efficiency of *AOC1* knockdown were examined in sw480 cells by qRT‐PCR and western blot analysis. **(B)** The effect of *AOC1* knockdown on cell proliferation was detected by Cell Counting Kit‐8 assay kits in sw480 cells. **(C)** Colony‐forming assay in *AOC1*‐depletion sw480 cells. CRC, colorectal cancer; *AOC1*, Amine Oxidase Copper Containing 1 mRNA: messenger RNA; qRT‐PCR, real‐time quantitative polymerase chain reaction; **p < 0.01, ***p < 0.001.

As mentioned above, *AOC1* was relatively highly expressed in the sw480 cell line. To further examine the effect of *AOC1* on the proliferation ability of CRC cells, sw480 cells were selected to knockdown *AOC1* expression with *AOC1*‐targeting shRNAs. shRNA-mediated depletion of *AOC1* was confirmed by qRT-PCR and western blotting assays (**Figure 6A**). The CCK-8 assay indicated that *AOC1* knockdown significantly inhibited CRC cell growth (**Figure 6B**). To further confirm this effect, a colony formation assay was also conducted (**Figure 6C**). These results confirmed the ability of *AOC1*-depletion to inhibit tumor cell proliferation.

### *AOC1* Knockdown Regulated the Migration Ability of CRC Cells *via* Inducing EMT

**Figure 7:**
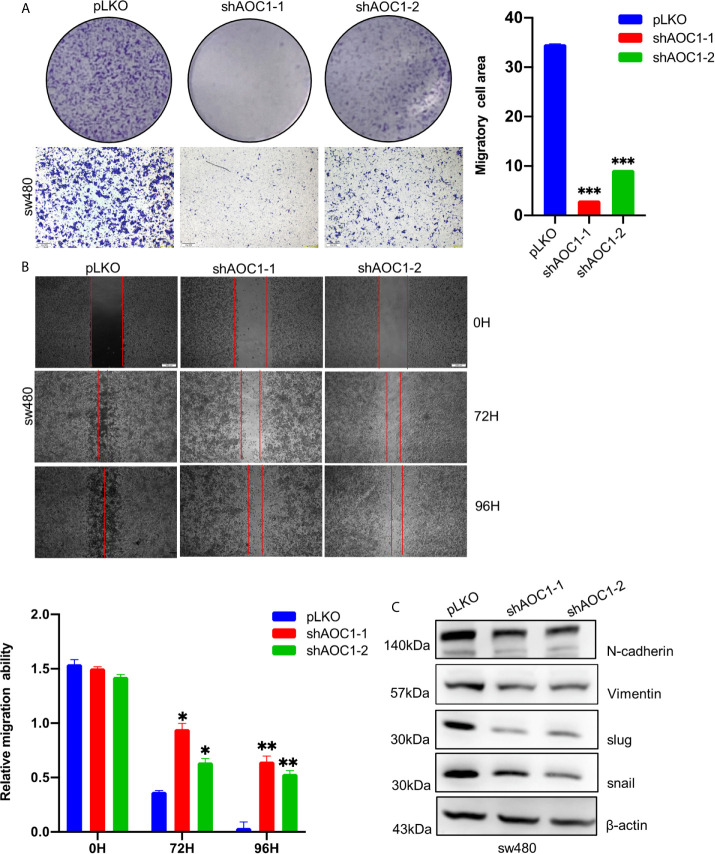
*AOC1* knockdown regulated the migration ability of CRC cells *via* inducing EMT. **(A)** The effects of *AOC1* knockdown on cell proliferation were detected by Transwell‐migration assays in sw480 cells (Scale bar:200um). **(B)** The migratory capacity of *AOC1*‐depletion or control sw480 cells were determined by wound‐healing assay. The extent of migration was determined at 72 h after the initial scratch wound (Scale bar:200um). **(C)** EMT related proteins: N‐cadherin, Vimentin, Slug, SNAIL were detected in *AOC1* – depletion sw480 cells by western blot. *AOC1*, Amine Oxidase Copper Containing 1; EMT, epithelial–mesenchymal transition; *p < 0.05, **p < 0.01, ***p < 0.001.

We further assessed the effect of *AOC1* knockdown on the migration ability of CRC cells using Transwell and wound healing assays. As shown in **Figure 7A**, *AOC1*-depletion in sw480 cells significantly inhibited migration ability compared with control cells by Transwell assay. Based on the Transwell test, an identical result was confirmed using a wound-healing assay(**Figure 7B**). Next, we investigated whether AOC1 affects the migration ability in CRC *via* inducing epithelial–mesenchymal transition (EMT) process. As shown in **Figure 7C**, we found that *AOC1*-depletion decreased the expression of N-cadherin and Vimentin by western blotting. Meanwhile, when AOC1 was silenced, the upstream transcription factors SNAIL and Slug were also significantly down-regulated.

### *AOC1* Knockdown Inhibited the Tumor Xenografts Growth *In Vivo*

**Figure 8:**
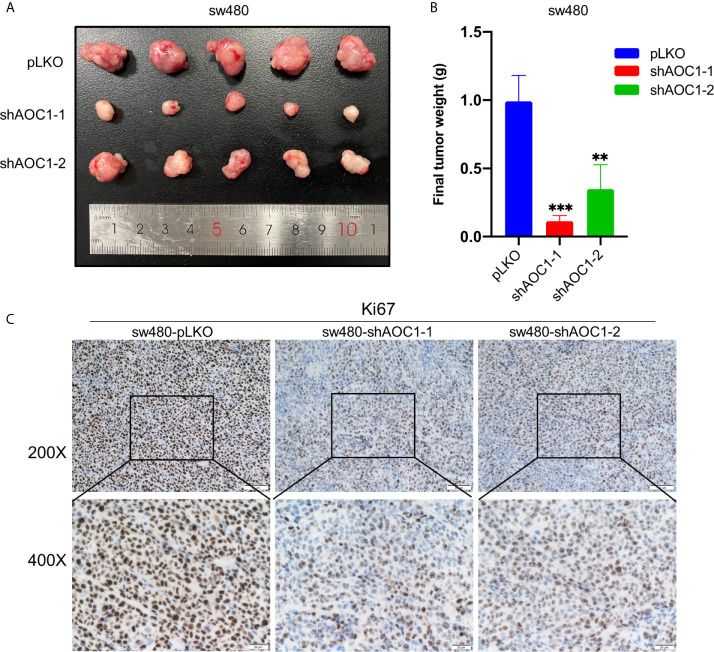
Knockdown of *AOC1* inhibited the tumor xenografts growth *in vivo*. **(A)** Representative of *AOC1-*knockdown sw480 xenograft tumors (n = 5). **(B)** The weight of the tumors in the different groups. **(C)** Immunohistochemical analysis of Ki67 expression in tumors from different groups (magnification: × 200, upper panels; × 400, lower panels). *AOC1*, Amine Oxidase Copper Containing 1; **p < 0.01, ***p < 0.001.

To explore the biological function of *AOC1-*depletion in tumor growth *in vivo*, a xenograft assay was performed by injecting paired tumor cells into nude mice. Consistent with the result of the cell proliferation assay *in vitro*, *AOC1-*knockdown sw480 xenograft tumors were dramatically smaller than the tumors derived from control sw480 cells, suggesting that knockdown of AOC1 significantly inhibited the tumor xenografts growth (**Figures 8A, B**). Furthermore, Ki67 staining of the xenograft tumors showed that *AOC1-*knockdown decreased the number of Ki67-positive cells in the xenograft tumors (**Figure 8C**). In summary, these results indicated that AOC1 played an oncogenic role in promoting tumor growth.

## Discussion

CRC has become a major global health concern with its high incidence rate and mortality rate. Interestingly, while we have witnessed a declining incidence trend over the past few decades in the elderly, the incidence rates for adolescents and young adults have been on a steady rise (15). Therefore, there is an urgent need to identify biomarkers and therapeutic targets to accurately predict the occurrence and progression of CRC.

Mammalian copper-containing amine oxidase (CAO), encoded by four genes (*AOC1* 1-4) and catalyzes the oxidation of primary amines to aldehydes, regulates many biological processes and is associated with many diseases including inflammation and histamine intolerance (16). *AOC1* encodes a metal-binding membrane glycoprotein that oxidizes putrescine, histamine, and related compounds. Amine oxidase is mainly involved in tumor growth inhibition and progression (17). Although previous reports revealed that *AOC1* affected the occurrence and development of gastric cancer (8) and Wilms tumors (8, 18), its role in colorectal cancer has not been elucidated. In this study, we first identified the biological functions of *AOC1 in vitro* and *vivo*, especially on proliferation and migration ability.

Firstly, we found that AOC1 was highly expression in tumor tissues both in COAD and READ, and AOC1 expression was statistically significant for predicting tumor clinical stage according to GEPIA database (p=0.0459). Then, we detected the expression of *AOC1* in CRC tissues and found that *AOC1* expression was significantly increased in human colorectal cancer tissues compared to normal tissues. In addition, the expression of *AOC1* was higher in liver metastatic tissues than in primary tumor sites and significant associated with liver metastasis (p < 0.001). Furthermore, with the advent of the era of organoid culture *in vitro*, we found that *AOC1* had higher expression in tumor organoids than in normal organoids by using this advanced technology, suggesting that it was highly located in the tumor epithelium. These results suggested that *AOC1* was involved in the tumorigenesis, development, and prognosis of human colorectal cancer. We then verified whether *AOC1* has the same aforementioned biological function in CRC tumor cells.

Secondly, we constructed an overexpression stable system and found that *AOC1* overexpression promoted the proliferation of CRC cells by cck8 and colony formation assays, as well as migration ability by transwell and wound healing assays. Interestingly, *AOC1* knockdown inhibited both the migration and proliferation ability of CRC cells. To further study the mechanism through which AOC1 affects the occurrence and development of CRC, as well as promoting tumor proliferation and metastasis, we found that AOC1 regulated the migratory ability of CRC cells by EMT pathway. EMT encompasses dynamic changes in cellular organization from epithelial to mesenchymal phenotypes, such as increased motility, which was associated with an invasive or migratory ability in CRC (19, 20). In this study, we found that the mesenchymal hallmarks N-cadherin and vimentin expression decreased in *AOC*1-depletion sw480 cells, moreover, the upstream transcription factors SNAIL and Slug were also decreased. Therefore, AOC1 promoted the development of CRC through epithelial-mesenchymal transition, as reported in a previous article (8). Therefore, targeting *AOC1* therapy by reverse the EMT process may be an effective and potential strategy for better prognosis in patients with CRC.

These results comprehensively demonstrated that high expression of *AOC1* was significantly associated with worse clinical outcomes, was an independent risk factor for poor prognosis, and promoted the aggressive phenotypes of CRC cells by inducing EMT. Moreover, *AOC1* is presumed to be a novel biomarker for predicting the prognosis of patients with CRC and an effective therapeutic target in clinical practice.

However, there were some limitations encountered in the present study. The sample size was relatively small, and the design was retrospective. Loss to follow-up was inevitable, and clinical significance needs to be verified in a larger sample. In summary, we demonstrated that *AOC1* promoted CRC progression and is significantly associated with poor clinical outcomes. It could be used as a new independent prognostic biomarker and a potential therapeutic target for the treatment of CRC. In the next study, We will compare the specificity and sensitivity of AOC1 and CEA to analyze whether AOC1 can replace or be used in combination with CEA to predict colorectal cancer and its prognosis clinically.

## Data Availability Statement

The original contributions presented in the study are included in the article/supplementary material. Further inquiries can be directed to the corresponding authors.

## Ethics Statement

The studies involving human participants were reviewed and approved by The Ethics Committee of Xinhua Hospital approved this study (No. XHEC‐D‐2021‐002). The patients/participants provided their written informed consent to participate in this study. The animal study was reviewed and approved by the Laboratory Animal Care and Welfare Committee of Xinhua Hospital (No. XHEC‐F‐2021‐057). Written informed consent was obtained from the individual(s) for the publication of any potentially identifiable images or data included in this article.

## Author Contributions 

PD and WX conceived and designed the study. FL performed experiments and wrote the manuscript. WO and WT analyzed the data. assisted with the statistical analysis. ZH and ZZ assisted with some experiments. WD, JF, and YZ collected the clinical data. CL supervised the work and data analyses. All authors contributed to the article and approved the submitted version.

## Funding

This work was supported by the Natural Science Foundation of Shanghai (20ZR1435000), National Natural Science Foundation of China (No.82000481), and the Shanghai Sailing Program (No. 20YF1429400).

## Conflict of Interest

The authors declare that the research was conducted in the absence of any commercial or financial relationships that could be construed as a potential conflict of interest.
